# Zr^4+^-Coordinated Highly Stretchable and Conductive Silk Fibroin/PPy Hydrogel for Flexible Wearable Sensing

**DOI:** 10.3390/polym18121502

**Published:** 2026-06-16

**Authors:** Mujin Yang, Qihan Jia, Shuang Wang, Haibo Wang

**Affiliations:** College of Biomass Science and Engineering, Sichuan University, Chengdu 610065, China; mujin_yang0412@163.com (M.Y.); qihanjia@163.com (Q.J.)

**Keywords:** wearable strain sensors, conductive hydrogels, silk fibroin, polypyrrole, zirconium ion

## Abstract

Conductive hydrogels are promising materials for fabricating flexible wearable strain sensors. However, their practical application remains limited by several challenges, including poor mechanical strength, unstable sensitivity, restricted stretchability, and poor structural durability. In this study, a zirconium-reinforced conductive hydrogel (PSPZr) with a dual chemical–physical cross-linked network was designed and developed. In the structural framework, polypyrrole-decorated silk fibroin (SF/PPy) functioned as a conductive reinforcing component, acrylamide and sulfobetaine methacrylate constituted the flexible polymer basis, and zirconium ions (Zr^4+^) acted as ionic cross-linkers to construct a dual cross-linked structure and improve mechanical stability. Due to the synergistic contributions of hydrogen bonding, ionic coordination interactions, and SF/PPy, the optimized PSPZr hydrogel exhibited a tensile strength of 166 kPa and a maximum strain 559%. Additionally, it achieved improved elasticity and reliable shape recovery. Furthermore, the optimized PSPZr hydrogel exhibited a broad working range, sensitivity with a gauge factor of 2.8, rapid response, recovery kinetics, and exceptional cycling stability over 1000 stretching–releasing cycles as wearable strain sensors. This performance enabled real-time and accurate monitoring of diverse human motions. Therefore, this study presents a feasible and versatile strategy for developing mechanically robust and electrically stable conductive hydrogel, providing a new pattern for advanced applications in wearable sensors.

## 1. Introduction

In recent years, the increasing demand for durable, intelligent, and portable electronic devices with high detection, capacity, and adaptive functionality has prompted the development of wearable materials, such as gels [1,2,3]. Wearable strain sensors are crucial components of smart wearable devices and other related devices, including supercapacitors and pressure sensors. They convert physical movements into electrical signals and enable motion monitoring and human–computer interaction [4,5,6,7,8]. Although existing methods for producing related polymer elastomers and flexible membranes mainly rely on the incorporation of functional materials, including multi-dimensional transition metal nitride (MXene) [9], electrically sensitive organic compounds [10] and metal nanowires [11], conductive gels have emerged as promising materials for wearable strain sensors owing to their synergetic advantages of excellent conductivity, outstanding flexibility, multi-functional modification potential, and other improved properties [12,13]. However, the development of gel-based materials remains a challenge, particularly in achieving the simultaneous integration of improved mechanical performance, long-term stability, and self-strengthening ability within a single system capable of meeting practical application requirements [14].

Conductive hydrogel is a promising sensing material that combines conductive elements and a hydrogel framework into its network structure [15]. Studies have shown that conductive hydrogel-based flexible sensors can effectively address the limitations of conventional conductive materials [16,17,18]. Among the various strategies developed, conductive polymers such as polyaniline (PANI) [19] and poly(3,4-ethylene dioxythiophene): poly (styrene sulfonate) (PEDOT:PSS) [20] have been widely integrated into hydrogel networks to enhance their electrical conductivity. In addition, carbon nanomaterials have been used as a conductive component for producing conductive hydrogels [21]. Moreover, metal-based conductive hydrogels [22] and low-toxic ionic conductive hydrogels [23] exhibit outstanding conductivity owing to their three-dimensional network structure. However, hydrogel-based flexible sensors are characterized by poor mechanical strength [24,25], low sensitivity [26], and the potential toxicity of certain conductive components, particularly some metal ions, thus limiting their application [27]. Polypyrrole (PPy), a widely used conductive polymer, has attracted considerable attention in hydrogel fabrication owing to its high electrical conductivity and ease of functionalization, making it an effective new component in hydrogel synthesis [28,29,30]. It can be combined with different macromolecules, such as alginate-gelatin [29], chitosan [31] and fibroin protein [32] to fabricate enhanced conductivity polymers. However, its excessive application can lead to brittleness in the resulting material. Silk fibroin (SF), a natural protein, can form a dense framework that endows hydrogels with excellent mechanical properties, low biological rejection, and adhesiveness to biological tissues. Several SF-based materials have been developed to enhance the physical properties and biocompatibility of various basic hydrogels [32,33,34]. Zirconium (Zr^4+^) could improve the mechanical strength of conductive hydrogels [35], which is a stable metal ion with low toxicity and can readily form coordination complexes with natural polymers [36,37], capable of building complexes with renewable polymers such as cellulose, collagen fibers [38], and sodium alginate. Compared with ions, including Cu^2+^, Mn^2+^, and Ca^2+^, Zr^4+^ can crosslink more effectively with the protein-based network structures incorporated into some hydrogels, thereby enhancing the physical performance of the materials [39,40].

In this study, a zirconium-reinforced conductive hydrogel with distinct mechanical characteristics, highly sensitive conductivity and excellent surface viscosity was prepared and used for wearable strain sensors. In this system, a conductive PPy-coated silk fibroin (labeled as SF/PPy) was produced through the self-polymerization of PPy on the surface of the SF. Then, acrylamide (AM) and sulfobetaine methacrylate (SBMA) were dissolved in an aqueous system as monomers for polymerization, and the SF/PPy was added to obtain a homogeneous solution system. Then, Zr^4+^ from zirconium oxychloride octahydrate was introduced into the system to enhance the mechanical strength of the 3D polymer networks, resulting in a zirconium-reinforced conductive hydrogel production, and a series of relevant characterizations and experimental tests were conducted. The results showed that the conductive hydrogel exhibited excellent tensile properties, stability, and accuracy in electrical signal testing. Therefore, the zirconium-reinforced conductive hydrogels are promising materials for manufacturing wearable devices and bioelectronics.

## 2. Materials and Methods

### 2.1. Materials

Acrylamide (AM), sulfobetaine methacrylate (SBMA), polypyrrole (PPy), silk fibroin (SF), zirconium oxychloride octahydrate (ZrOCl_2_·8H_2_O), *N*,*N*′-methylenebisacrylamide (MBA), ammonium persulfate (APS), and *N*,*N*,*N*′,*N*′-tetramethylethylenediamine (TEMED) were provided by Aladdin (Shanghai, China). All chemicals were used as acquired without further treatment.

### 2.2. Preparation of the Polypyrrole-Coated Silk Fibroin (SF/PPy)

Preparation of the SF/PPy: a 1% (*v*/*v*) dilute hydrochloric acid solution was prepared for pretreatment, and 1.74 g of SF was added to 50 mL of the liquid under continuous magnetic stirring at 15 °C for 10 min until the solution turned transparent. After that, 0.05 g PPy and 0.08 g APS were added to the solvent under continuous magnetic stirring at 15 °C for 1.5 h, until a dark green, flocculent, turbid solution was produced. Subsequently, the mixture was washed at 8000 rpm for 10 min to discard the supernatant. The precipitate was washed by adding 50 mL of deionized (DI) water, shaking thoroughly for 5 min, followed by centrifugation at 8000 rpm for 10 min. This washing process was repeated twice. Finally, the samples were dried at 80 °C to get pure SF/PPy powder, which could be sealed and stored at 5 °C in a constant temperature and humidity chamber.

### 2.3. Synthesis of the PAM-Co-SBMA/SF-PPy/Zr Hydrogels (PSPZr Hydrogels)

Zirconium-reinforced conductive hydrogels: weighing 2.0 g AM, 0.25 g SBMA, and 4 mg MBA with an analytical balance, and 0.5 g pure SF/PPy was added to 25 g of DI water and sonicated for 20 min to homogenize for the next step of polymerization. Then, different amounts of SF/PPy solution (weight ratios of 0 wt%, 20 wt%, 40 wt%, and 60 wt%) and DI water were mixed to form a solution weighing 6.0 g in total. Other materials were added into the liquid under continuous magnetic stirring at 15 °C for 20 min until a dark gray solution was formed. After that, add 25 mg ZrOCl_2_·8H_2_O and 20 mg APS with a quick stirring. Then, the precursor solution was ultrasonically treated for 5 min to remove the bubbles. Eventually, 50 μL TEMED was added to the solution to initiate the reaction. The solution was instantly moved to a PTFE mold and was polymerized under 15 °C for 1.5 h, until a zirconium-reinforced conductive hydrogel was produced. The samples then received hermetic sealing and refrigerated storage at 5 °C, which were later equilibrated for 30 min under standard conditions before testing. The specific formulation of PSPZr hydrogel is shown in Table 1.

### 2.4. Characterizations

The chemical structures of PSPZr hydrogels were analyzed by Flourier transformation infrared spectroscopy (FTIR), which was conducted from 500 to 4000 cm^−1^ at a resolution of 4 cm^−1^ using a Nicolet iS5 FTIR spectrometer (Nicolet, Thermo Fisher, Waltham, MA, USA). The surface structure and chemical features were tested by X-ray photoelectron spectroscopy (XPS) (Nexsa, Thermo Fisher, Waltham, MA, USA). The X-ray diffraction (XRD) patterns of the hydrogels were confirmed with X-ray diffractometry (XRD, Rigaku Smart Lab, Akishima, Japan) by Cu Kα radiation at 1.54060 Å. The morphologies of the samples were investigated by Scanning electron microscope (SEM) (TSM-7500F, JEOL, Tokyo, Japan).

The mechanical features of the samples were assessed by a tensile testing machine (Instron 5967 universal, Instron Electron Instrument, Norwood, MA, USA) under room temperature conditions. Three different sets of hydrogels from SF/PPy-0 to SF/PPy-60 were prepared for testing in advance. All samples were sealed in bags right after preparation and stored in a 5 °C environment to secure the hydration state, which required a 30-min recovery to the testing temperature. The related tests were conducted under standard conditions. A series of single and cyclic tensile tests was conducted at 50 mm per minute on dumbbell-shaped specimens, which were 30 mm × 4.0 mm × 3.5 mm in dimensions.

The sensing performance was tested using rectangular samples (SF/PPy-40). Copper wires with clamps at each end were attached to both ends of the samples. The various motions of the human body, such as the fingers, wrist, knees, neck, and ankles, were monitored by analyzing the instant resistance changes of the sensors.

## 3. Results and Discussion

### 3.1. Polymerization of the PSPZr Hydrogels

**Figure 1:**
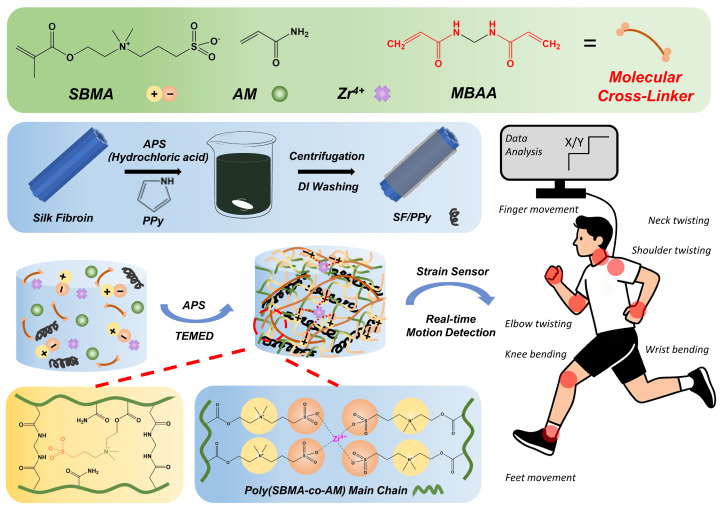
Schematic of the preparation of PSPZr hydrogels.

The synthetic procedure to prepare zirconium-reinforced conductive hydrogels is illustrated in Figure 1. The resulting products exhibited a deep gray appearance and a moist surface. PPy was incorporated to construct an electron-rich conductive network that enabled the material to conduct electricity. As illustrated in Figure 1, a conductive SF/PPy combining SF with the PPy conductive structure was formed, which was then introduced to produce the hydrogels. After that, AM and SBMA were used as monomers to construct a three-dimensional structure with MBA as the crosslinker, catalyzed by APS and initiated by TEMED. Meanwhile, the Zr^4+^ ions reinforced the polymer network through ionic interactions, thereby enhancing the mechanical properties of the hydrogels.

**Figure 2:**
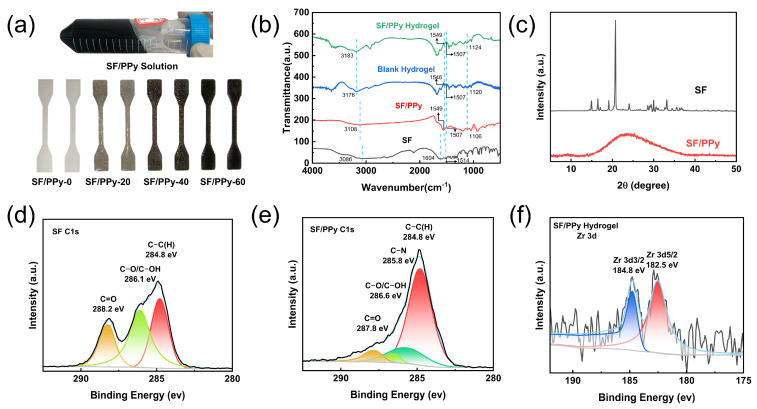
Samples and chemical properties of the PSPZr hydrogels. (**a**) The photograph of the SF/PPy contained in a 50 mL tube and the PSPZr hydrogels with different SF/PPy from 0 wt% to 60 wt%. (**b**) The FTIR spectra of SF, SF/PPy, Blank hydrogel, and SF/PPy hydrogel (PSPZr hydrogels). (**c**) XRD spectra of SF and SF/PPy. XPS high-resolution C1s spectra of the (**d**) SF, (**e**) SF/PPy (the color-coding is red (C−C(H)), green (C−O/C−OH), yellow (C=O), and deep green (C−N)). XPS high-resolution Zr 3d spectra of the (**f**) SF/PPy Hydrogel (the color-coding is red (Zr 3d5/2), blue (Zr 3d3/2)).

As shown in Figure 2a, the color of the PSPZr hydrogel exhibited progressive darkening with increasing SF/PPy content. This color change was attributed to the SF/PPy incorporated into the hydrogel. As shown in Figure 2b, FTIR revealed the chemical structures of the PSPZr hydrogel. The characteristic peaks of SF at 1604 cm^−1^ (amide I, *β*-sheet) and 1514 cm^−1^ (amide II) dramatically decreased in the SF/PPy material [41,42,43], while the peaks representing pyrrole appeared at approximately 1550 cm^−1^ (C=C stretching) and 1106 cm^−1^ (C-N stretching), both indicating the successful in-situ oxidative polymerization of pyrrole and PPy coating onto the SF [31,41,44]. Moreover, the N-H/O-H peak of pure SF, located at 3078 cm^−1^, shifted to 3108 cm^−1^ after PPy incorporation. This shift confirmed the existence of new hydrogen bonds between the -NH- groups of PPy and the -OH/-NH- groups of SF [28]. Further, the FTIR spectrum of the SF/PPy hydrogel displayed characteristic peaks of the PPy that were slightly different from those of the control group, providing additional evidence for the successful incorporation of SF/PPy into the hydrogel system. The FTIR spectrum of the pure hydrogels exhibited several characteristic absorption bands that were also observed in the SF/PPy hydrogel, which could be attributed to the relatively low absorbance intensity of the SF/PPy mixture compared with the absorption of the hydrogel. XRD revealed the crystalline structures of SF and SF/PPy. As shown in Figure 2c, the 2θ peak of SF at 20° confirmed its amorphous structure [45]. Additionally, PPy exhibited a broad peak at 25° [44,46]. In comparison, the PPy-decorated SF exhibited a wide peak in the range of 15.0° to 35.0°, which further verified the introduction of PPy [44].

XPS was used to analyze the chemical structures of the SF/PPy. Figure 2d,e shows the peaks of C1s and O1s. The C/O ratios of SF/PPy and SF were 4.61 and 0.61, respectively, indicating the successful deposition of carbon-rich PPy on the SF surface and providing further evidence for the formation of the SF/PPy composite. Moreover, XPS C1s spectra of SF (Figure 2e) and SF/PPy (Figure 2f) revealed characteristic peaks, corresponding to C−C(H), C−O/C−OH, C=O, and C-N, respectively. As shown in the deconvoluted spectra, the SF exhibited a dominant C−C/C−H peak at 284.8 eV, which was a characteristic of the protein backbone structure. After PPy was introduced to the SF, the SF/PPy displayed significantly enhanced intensity of the C−C/C−H band at approximately 284.8 eV and a visibly decreased peak (C−O/C−OH) at 286.3 eV. This observation could indicate that the pyrrole rings were deposited on the surface of SF by reacting with hydroxyl groups originating from tyrosine and serine of SF. Notably, a new C-N related peak appeared at 285.6 eV in the SF/PPy spectrum, which was one essential structure of PPy, further confirming the successful in-situ oxidative polymerization of pyrrole on the SF scaffold and the formation of strong interfacial interactions between the conductive polymer and the protein matrix. The Zr 3d spectrum in Figure 2f confirms the Zr^4+^ in the SF/PPy hydrogel. Two specific peaks located at 182.5 and 184.8 eV were assigned to Zr 3d5/2 and Zr 3d3/2, respectively, resulting from spin–orbit splitting of the Zr 3d orbital. The binding energy of Zr 3d5/2 was in the range of 180.0 eV (Zr metal) and 182.2 eV (zirconia), further confirming the existence of zirconium in the form of Zr^4+^ ions. This observation could be attributed to chemical bonding between the Zr-ion core and the SF/PPy component, further confirming the coordination mechanism of Zr^4+^ [47].

**Figure 3:**
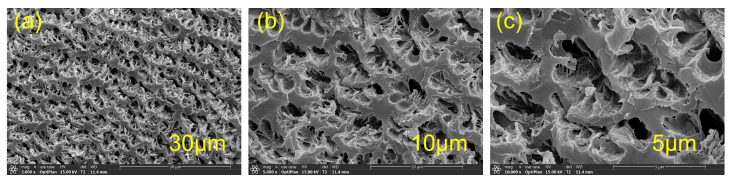
SEM images of PSPZr hydrogels with 40 wt% SF/PPy being fractured by quenching after liquid nitrogen flash freezing and freeze-dried at different magnifications of (**a**) 30 μm, (**b**) 10 μm, and (**c**) 5 μm.

The morphologies of lyophilized PSPZr hydrogels were observed using SEM. As presented in Figure 3, the PSPZr hydrogel exhibited a closely packed porous morphology, and this dense 3D structure enhanced the mechanical performance of the hydrogel [32].

### 3.2. Mechanical Features

**Figure 4:**
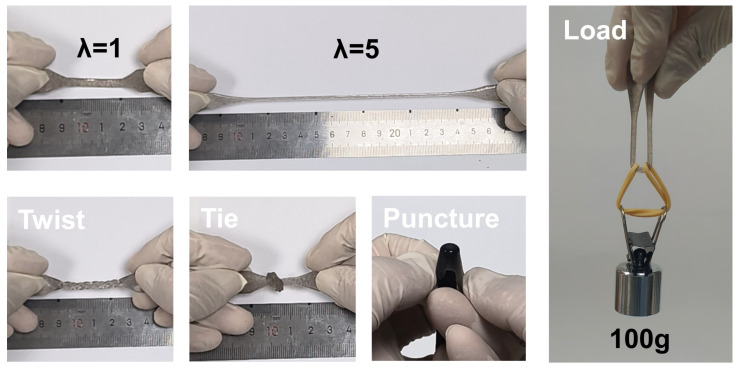
Snapshots of PSPZr hydrogels under external physical influences (stretching, tying, twisting, and loading–relaxing, and puncturing).

Mechanical properties are critical for wearable sensors, and outstanding mechanical durability enables sensors to respond to external forces, thereby providing the PSPZr conductive hydrogel with impressive potential for sensing different motion signals. As shown in Figure 4, PSPZr hydrogels maintained their structural integrity under stretching, twisting, and knotting. The SF/PPy-40 hydrogel could be stretched to 500% without structural damage. The stretched sample can recover rapidly to its initial length after the external stress is released. The testing results of the SF/PPy-40 lifting a 100 g weight also confirmed excellent mechanical endurance. Furthermore, the sample exhibited great resistance against puncturing led by external presses, which could return to the initial state once the force applied was removed.

**Figure 5:**
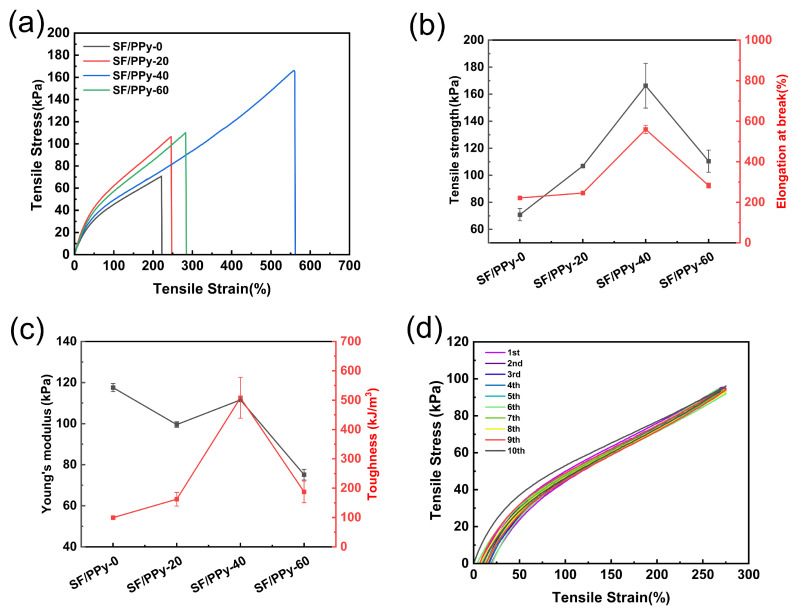
Mechanical properties of hydrogels: (**a**) Stress-strain curves for tensile samples based on PSPZr hydrogel; (**b**) Maximum tensile strength and elongation at break for varied SF/PPy contents; (**c**) The Young’s modulus and toughness for varied SF/PPy contents. (**d**) Curves of the total consecutive tensile loading-releasing tests for SF/PPy-40 under strain.

Tensile tests were conducted to further evaluate the mechanical properties of the PSPZr hydrogels with varying SF/PPy contents, as seen in Figure 5. As shown in Figure 5a,b, as the amount of SF/PPy increased from 0 wt% to 40 wt%, the tensile strain increased from 221 ± 7.25% to 559 ± 19.98%, tensile strength increased from 71 ± 4.52 kPa to 166 ± 16.55 kPa, the Young’s modulus was calculated from 0 to 1% strain, which decreased from 117 ± 2 kPa to 99 ± 1.37 kPa and then increased to 112 ± 0.14 kPa, and toughness increased from 99.6 ± 4.9 kJ/m^3^ to 508.1 ± 69.6 kJ/m^3^ (Figure 5c). These results showed that the SF/PPy-40 hydrogel improved mechanical properties compared with the control group without SF/PPy. This enhancement could be attributed to the enhanced effect of the Zr^4+^ cross-linking and the filled component SF/PPy. The increased SF content effectively facilitated transfer stress and external energy absorption, thereby enhancing deformation resistance and improving the mechanical properties of the PSPZr hydrogel [48]. In addition, PPy binds with SF through hydrogen bonding. However, further addition of SF/PPy to the hydrogel reduced toughness and strength caused by excessive crosslinking [49]. Therefore, SF/PPy-40 hydrogel exhibited the most favorable tensile mechanical properties, which surpassed those reported for other conductive hydrogels (Zheng et al., >500% [28], Niu et al., 94.1 ± 6.4 kPa [48]). The elasticity of the PSPZr hydrogel was confirmed by tensile cycling tests. As shown in Figure 5d, the stress–strain curves of the SF/PPy-40 hydrogel held a tensile capacity up to 275%. All loading–unloading cycles displayed negligible hysteresis behaviors, revealing outstanding elastic recovery. Furthermore, cyclic tensile curves of SF/PPy-40 received detailed analysis and demonstrated high elasticity and recovery behaviors. The hysteresis energy (Uhys) was 15.63 kJ/m^3^ during the first loading-unloading cycle, and a distinct hysteresis loop was observed, indicating that most of the material softening and hysteresis occurred during the first cycle. Moreover, the stress–strain curves during the 2nd to 10th cycles nearly overlapped, implying that the structure of the hydrogel reached a stable state [50]. This impressive energy dissipation endowed the PSPZr hydrogel with sufficient toughness in resisting stress.

### 3.3. PSPZr Hydrogel-Based Sensors and Performance

**Figure 6:**
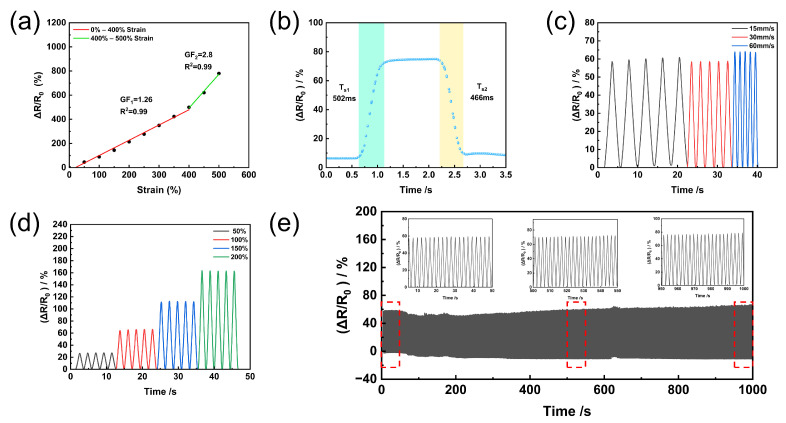
Strain sensing performance of the sensor made up of SF/PPy-40 hydrogel. (**a**) Relative resistance changes of SF/PPy-40–40 strain sensor as a function of set strain (0–700%). (**b**) Response time and recovery time of SF/PPy-40–strain sensor during the loading-unloading cycle. (**c**) Tests of a series of 100% strains loading-unloading cycles at different frequencies. (**d**) Tests of a series of periodic loading strains from 50% to 200%. (**e**) Cycling stability measurements of SF/PPy-40–strain sensor at 100% strain during the period of 1000 cycles.

Given their outstanding mechanical performance and excellent stretchability, the SF/PPy-40 hydrogels were further characterized for practical application scenarios, as shown in Figure 6. The SF/PPy-40 hydrogels exhibited a high-performance conductivity enabled by the uniform distribution of Zr^4+^ [51], while the PPy-coated SF surface provides an effective conduction pathway. Upon external force-induced stretching, the ion-transport pathways became narrower and longer, increasing electrical resistance [52]. In addition, the cross-linked PPy coating SF could undergo physical deformation, which could alter the transmitting routines for its delocalized π electrons moving in conjugated π bonds, thereby resulting in a corresponding change in electrical resistance. To further quantitatively analyze the sensitivity of the SF/PPy-40 hydrogel sensor, the gauge factor (GF) was determined using the equation below:

$$\mathrm{GF}=\frac{{\Delta R/R}_{0}}{\varepsilon }\;$$

R_0_ and R (Ω) represent the resistance of the sensor without and with applied stretching, and ε (%) represents the applied strain. As shown in Figure 6a, the GF for strain ranges of 0–400% and 400–500% were calculated to be 1.26 and 2.8, respectively. The recent conductive hydrogel strain sensors’ sensing properties are shown in Table 2. The sensitivity across the wide strain range remained stable, even in the high-strain region of 400–500%, indicating its suitability for large-range strain-sensing applications. Moreover, the resistance changes induced by strain are verified to be an excellent linear fit (R_1_ = 0.99, R_2_ = 0.99), leading to a simplified calibration procedure and improved signal acquisition accuracy and reliability.

As shown in Figure 6b, the gel sensor exhibited a response time of 502 ms and a recovery time of 466 ms under external stretching and releasing, respectively. This outstanding performance enables strain sensors to meet the requirements of high sensitivity over a broad range of strains and frequencies. As demonstrated in Figure 6c, the index features of SF/PPy-40 hydrogel remained stable and orderly in resistance to various frequencies, indicating excellent kinetic response ability. Figure 6d. presents the loading–unloading behavior of SF/PPy-40 hydrogel over a strain range of 50% to 200%. The sensor showed distinct and repeatable resistance changes to the cyclic test, small and high strains. Further cyclic testing of the SF/PPy-40 hydrogel sensor was performed with a series of cyclic stretching-releasing in Figure 6e, which eventually showed a gradual increase from 50% to 90% in relative resistance change, confirming the sensing performance was steady for 1000 cycles with no significant decay. This phenomenon indicated that the sensor performed well in cyclic loading-unloading tests, with remarkable cyclicity and durability, which could be attributed to improved contact between conductive networks and stable structural adaptation of the sensing material. In summary, SF/PPy-40 hydrogel exhibited high conductivity, a wide detection range, excellent repeatability, rapid response and recovery, and impressive endurance, indicating its strong potential for practical sensing applications.

### 3.4. Real-Time Exhibition as Wearable Strain Sensors

**Figure 7:**
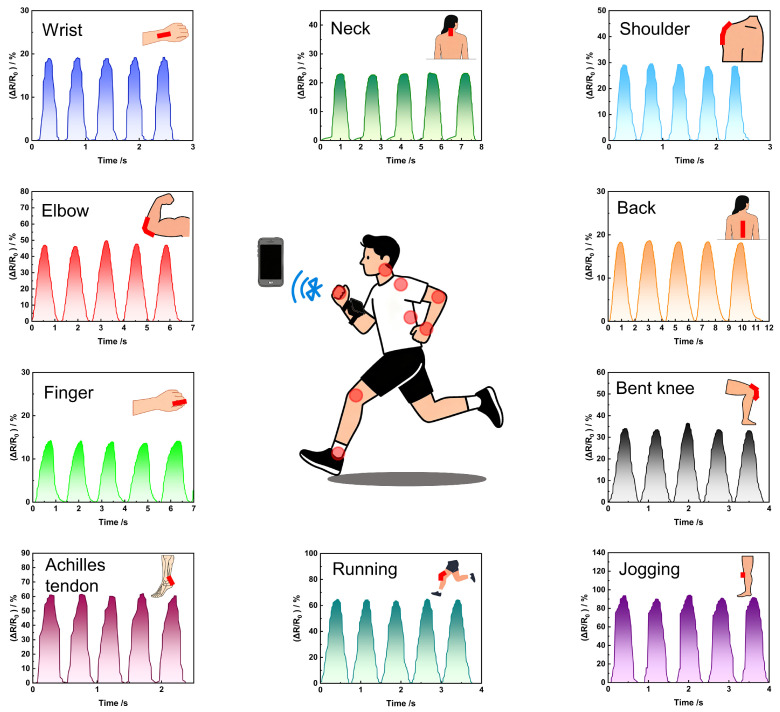
Illustration of PSPZr hydrogel sensor applied in human motion monitoring and demonstration of conductive hydrogels as E-skin. The relative current changes of the sensor response to wrist rotation, neck twisting, shoulder twisting, elbow twisting, back bending, finger movement, knee bending, ankle rotation, running, and jogging.

The PSPZr hydrogel with excellent mechanical and conductive properties was used to fabricate a flexible sensor for monitoring various human daily movements. The expanded strain-working range of the hydrogel-based sensor facilitates tracking of human movement. As shown in Figure 7, the SF/PPy-40 hydrogel was attached directly to the elbow to evaluate its ability to monitor elbow bending at different angles by recording the corresponding ΔR/R0 signals. In addition, finger-bending tests showed that ΔR/R_0_ of the sensor gradually increased as the tested finger bending increased from 0° to 90°. Distinct phase changes in ΔR/R_0_ corresponding to the stretching of the SF/PPy-40 hydrogel were observed during finger movement. The data exhibited a constant ΔR/R_0_ value when the finger was maintained at a certain bending angle, indicating stable sensing performance of the hydrogel. As the tested finger gradually returned to the initial straight state, the ΔR/R_0_ ratio of the sensor recovered to the original value, demonstrating good repeatability. As shown in Figure 7, the real-time resistance fluctuations of the strain sensor attached to the knee joint during running, walking, and jogging were further recorded to be stable and repeatable, which verified the PSPZr sensor’s capability for exercising movement detection. In addition, the changes in the hydrogel sensor’s ΔR/R_0_ value during the movement of more flexible and versatile joints, such as the neck, wrist, and ankle, were investigated. Using the wrist joint as an example, the ΔR/R_0_ response exhibited stability, periodicity behavior, and repeatability during circumferential movement of the wrist joint, which demonstrated the potential of PSPZr sensors in monitoring highly flexible joint movements. In conclusion, the sensor constructed on the basis of PSPZr hydrogel could be taken as a potential platform for wearable electronics detecting different human body movements.

## 4. Conclusions

In this study, a stretchable and conductive hydrogel with dual-network structures, reinforced by Zr^4+^ ions and PPy-coated SF, was successfully produced. Zr^4+^ was added as ionic points for physical cross-linking, and SF/PPy was added as a conductivity and mechanical performance-enhancing material in the 3D structure formed by PAM and SBMA. This modification endowed PSPZr hydrogel with excellent mechanical properties (166 kPa, 559%) and outstanding sensing performance. Considering the outstanding performance of the hydrogel, a wearable strain sensor was further produced. When the sensor was applied to monitor human movements, it generated clear and stable real-time resistance response signals for various movements, demonstrating reliable sensing performance. In summary, the PSPZr hydrogel demonstrated excellent conductivity and stability, indicating its considerable potential for human motion detection in wearable soft ionic conductive hydrogel sensors.

## Figures and Tables

**Table 1:** The composition of PSPZr hydrogels.

Sample	SF/PPy (mg)	AM (g)	SBMA (g)	ZrOCl_2_·8H_2_O (mg)	APS (mg)	MBA (mg)	TEMED (μL)	DI Water (g)
SF/PPy-0	0	2.0	0.25	25	20	4	50	6.00
SF/PPy-20	33	2.0	0.25	25	20	4	50	5.97
SF/PPy-40	65	2.0	0.25	25	20	4	50	5.94
SF/PPy-60	98	2.0	0.25	25	20	4	50	5.90

**Table 2:** Summary of recent conductive hydrogel strain sensors’ sensing properties.

Materials	Linear Sensing Range (%)	Guage Factor	Reference
CHs	0–100	0.10	[50]
P(AA-LMA)CTAB-Zr^4+^-Eq hydrogels	0–100	2.7	[51]
	100–200	1.3	
G_0.5_PL_1.0_ hydrogels	0–200	0.78	[53]
	200–400	2.18	
	400–500	3.71	
PSPZr hydrogels	0–400	1.26	This work
	400–500	2.80	

## Data Availability

The original contributions presented in this study are included in the article. Further inquiries can be directed to the corresponding authors.
